# Are telemedicine systems effective healthcare solutions during the COVID-19 pandemic?

**DOI:** 10.1016/j.jtumed.2021.02.009

**Published:** 2021-03-18

**Authors:** Gopi Battineni, Giulio Nittari, Ascanio Sirignano, Francesco Amenta

**Affiliations:** a Telemedicine and TelePharmacy Centre, School of Medicinal and Health Products Sciences, University of Camerino, Camerino, Italy; b Legal Medicine Division, School of Law, University of Camerino, Camerino, Italy; c Research Department, International Radio Medical Centre (C.I.R.M.), Rome, Italy

Dear Editor,

On 9 January 2020, China's Centres for Disease Control and Prevention (CDC) reported that a novel coronavirus causing a severe acute respiratory syndrome (SARS-CoV-2) had been identified as the causative agent of an aggressive respiratory disease, later referred to as coronavirus disease 2019 (COVID-19).^1^ As of 18 January 2021, there have been over 90 million reported cases of COVID-19 and the virus has been responsible for nearly 2.5 million deaths.^2^ The COVID-19 emergency has required continued contingency plans, making it necessary to both rethink the current approach to healthcare as well as how to adapt to the emerging needs of healthcare in the context of a pandemic. We have learned how to mitigate the spread of the virus by implementing social distancing measures, enforcing proper mask compliance, and reducing face-to-face contact in a health setting unless absolutely necessary. Community spread from the virus must be prevented to minimise the risks of infection for health professionals. In this respect, essential telemedicine services may help safeguard public health in significant ways.^3^

The term ‘telemedicine’ was introduced in the 1970s by Thomas Bird, an American who used it to refer to the delivery of medical assistance via telecommunication devices without a physical meeting between doctors and patients.^4^ In simple words, telemedicine can be defined as the provision of healthcare for people in remote areas when the providers and receivers of assistance are not in the same place. There is a substantial body of scientific literature that specifically discusses the potential of telemedicine. Notably, telemedicine encompasses the use of video consultations, electronic prescriptions, and remote management and monitoring of symptoms and vital parameters. This can become an indispensable response, especially in disasters like COVID-19 and other high-risk situations involving public health.

No telemedicine programme can be created and activated overnight; however, the health systems of countries like the United States, United Kingdom, Germany, and Canada have already invested in telemedicine, thereby ensuring that COVID-19 patients will receive the treatment and assistance they need.^5^ In Italy, the use of telemedicine in the pandemic context has become a topic of interest, as evidenced by the documents provided by the National Institute of Health (NIH) (Istituto Superiore di Sanità, ISS).^6^ There have been national guidelines on the use of telemedicine since 2012, but there are still several obstacles to implementation such as service costs and the lack of regulations.^7^ This hinders the effective use of these tools throughout the national territory. All activities involving a citizen's personal and health data, which are essential for the provision of telemedicine services, are subject to the secure processing of sensitive data carried out through electronic tools. Thus, the fundamental methods and solutions have to ensure data confidentiality, integrity and availability; therefore, in any case, telemedicine has to be adopted as per the government rules.

It is important to ensure the responsible use of telemedicine systems, and this can only be possible when the systems are used with better care and in the interest of public safety. The implementation of telemedicine should focus on monitoring patients with mild or asymptomatic cases, which then reduces hospital overcrowding. Accordingly, the possibility that crowding will be lessened also reduces virus spread, not only for patients but also for healthcare personnel.^8^ The usefulness of telemedicine solutions during the COVID-19 pandemic has already been confirmed in the case of remote areas in China, where the mortality rates and the virus spread were significantly higher than in cities with good healthcare systems.

New pandemic events created unique challenges in the provision of healthcare services. Although telemedicine does not present a solution to all problems, it is an important healthcare resource for people who are highly stressed. This includes citizens living in areas where medical services are difficult to access for geographic or other reasons, along with seafarers, who, when at sea, are essentially remote individuals.^9^ The provision of telemedicine should also comply with the ethical and legal standards attached to the medical profession, although relevant gaps still exist between the potentialities of technology and legislation/regulations.^10^ In terms of user responsibilities, it is important to highlight ethical issues concerning telemedicine.

With good reason, the current COVID-19 emergency is a call to action. The political responses of governments should include clear provisions on different fronts to evaluate their telemedicine measures as well as their interconnected implications. Subsequently, after the emergency phase, it will also be important to determine if these new approaches can help establish best practices in medicine that respect both the patients and organisations. Once telemedicine has been introduced to the Italian environment, if it is uniformly well-regulated throughout the national territory, it can play a significant role in responding to the current pandemic as well as any future such crises. Telemedicine also presents a solution to the anticipated future lack of healthcare personnel and to the vulnerability of the National Health Service (NHS) as exposed by the COVID-19 pandemic. It will then become necessary to think about and outline specific catalytic experiences—which are understood as contexts of dialogue and discussion, of education and learning as well as of regulations— to try to transform the challenges posed by the emergency into opportunities for improvement.

*Telemedicine has consistently become more important over the past few decades; the COVID-19 new pandemic has only underscored the importance of its immediate adoption. Furthermore, reports indicate that between February 2020 to June 2020, telemedicine consultations sharply increased from 36 million to 200 millionand are soon projected to reach 1 billion by end of 2020.**^11^* *With telemedicine's present direction and widespread adoption, it can disrupt current well-being frameworks, as well as manage costs and care delivery, thus creating a stage for vastly unique medical services in the future.*

## Source of funding

This work was supported in part by the ITF Trust (London, UK) grant No. 1508/2020.

## Conflict of interest

The authors have no conflict of interest to declare

## Ethical approval

The authors confirm that this editorial had been prepared in accordance with COPE roles and regulations. Given the nature of the editorial, the IRB review was not required.

## Authors' contributions

GB and GN conducted research, provided research materials, and collected and organised data. GB, AS and FA wrote the initial and final draft of the article. All authors have critically reviewed and approved the final draft and are responsible for the content and similarity index of the manuscript.
